# Rapid and PCR-free DNA Detection by Nanoaggregation-Enhanced Chemiluminescence

**DOI:** 10.1038/s41598-017-14580-w

**Published:** 2017-10-25

**Authors:** Renu Singh, Alexandra Feltmeyer, Olga Saiapina, Jennifer Juzwik, Brett Arenz, Abdennour Abbas

**Affiliations:** 1 Department of Bioproducts and Biosystems Engineering, University of Minnesota Twin Cities, St. Paul, MN USA; 2 USDA Forest Service, Northern Research Station, St. Paul, MN 55108 USA; 3 Department of Plant Pathology, University of Minnesota, St. Paul, MN USA

## Abstract

The aggregation of gold nanoparticles (AuNPs) is known to induce an enhancement of localized surface plasmon resonance due to the coupling of plasmonic fields of adjacent nanoparticles. Here we show that AuNPs aggregation also causes a significant enhancement of chemiluminescence in the presence of luminophores. The phenomenon is used to introduce a rapid and sensitive DNA detection method that does not require amplification. DNA probes conjugated to AuNPs were used to detect a DNA target sequence specific to the fungus *Ceratocystis fagacearum*, causal agent of oak wilt. The hybridization of the DNA target with the DNA probes results in instantaneous aggregation of AuNPs into nanoballs, leading to a significant enhancement of luminol chemiluminescence. The enhancement reveals a linear correlation (R^2^ = 0.98) to the target DNA concentration, with a limit of detection down to 260 fM (260 × 10^−15^ M), two orders of magnitude higher than the performance obtained with plasmonic colorimetry and absorption spectrometry of single gold nanoparticles. Furthermore, the detection can be performed within 22 min using only a portable luminometer.

## Introduction

Gold nanoparticles (AuNPs) are widely used nanomaterials for their excellent electric, catalytic, and optical properties^1^. Unique chemical and physical properties of AuNPs include high extinction coefficients in the visible region, alterable surface plasmon resonance (SPR) absorption, simple preparation, and easy functionalization. Therefore, AuNPs-based assays offer broad analytical capabilities and have already found multiple applications in detection of ions^2,3^, small molecules^4,5^, proteins^6,7^, DNA^8,9^, and cancerous cells^10,11^. Target hybridization with a labeled oligonucleotide probe is one the most widely used methods for detection of sequence-specific DNA nowadays^12,13^. The use of gold nanoparticles as labels of oligonucleotide probes in DNA detection have received a significant amount of interest due to the advantages of the AuNPs-based assays compared to assays where other labels are used (e.g., radioactive, fluorescent, chemiluminescent, and enzymatic labels). DNA detection with use of AuNPs labels often confers high sensitivity, speed and simplicity to the detection^14^. Up-to-date techniques that have been developed to monitor AuNPs-mediated DNA hybridization, include colorimetric, conductometric and fluorometric assays^15–17^, surface-enhanced Raman spectroscopy^18^, laser diffraction^19^, surface plasmon resonance^20^. Among these techniques, biodetection that exploits the localized surface plasmon resonance (LSPR) properties of AuNPs seems to have particular potential and perspectives^21^. Furthermore, the assembly or aggregation of gold nanoparticles results in the enhancement of LSPR due to plasmonic coupling of adjacent gold nanoparticles when the inter-particle distance is below 20 nm^22^. When the aggregation is significant (>30%), the phenomenon leads to a color change of the nanoparticle solution^23^. These properties have been previously harnessed by our group and others to develop detection technologies by analyzing the shift in the LSPR wavelength using a UV-visible spectrometer^24^, the Raman shift in surface-enhanced Raman scattering^25,26^, or by developing a colorimetric assay for visual assessment^27^.

Nucleic acid detection using plasmonic nanoparticles decorated with oligonucleotides probes has been investigated by many research groups^28,29^. However, when plasmonic colorimetry or absorption spectroscopy are used as transduction methods, the limit of detection (LOD) is typically limited to the nanomolar level^30,31^. When fluorophore or bar-code tagged nanoparticle techniques are used, the LOD reaches the femtomolar level but the analysis time is dramatically increased^8,32^.

Here, we show that the aggregation of gold nanoparticles not only enhances the plasmonic field but also leads to a significant enhancement of the chemiluminescence signal of luminophores. The phenomenon is used to introduce a sensitive and rapid DNA detection assay that can be performed in less than 22 min and only requires a hand-held or portable luminometer.

Chemiluminescence (CL), light emission induced by a chemical reaction, has been an attractive analytical tool for detection and quantification with a wide variety of applications due to its quick response, high sensitivity, low background noise, wide linear dynamic range, and the availability of hand-held luminometers^33,34^. In CL systems, oxidation of luminol is typically paired with other chemicals such as H_2_O_2_, K_3_Fe(CN)_6_, NaHCO_3_-H_2_O_2_^35–37^. For chemiluminescence to happen in luminol-H_2_O_2_ system, the presence of hydrogen peroxide as activator and a catalyst (e.g., iron, potassium ferricyanide or potassium periodate) is important. However, in the absence of a catalyst, the CL reaction may still occur but the signal is relatively weak. In alkaline solutions, luminol and hydrogen peroxide undergo the following transformations: firstly, luminol is oxidized by the hydroxide ion to the luminol dianion (L^•−^); then oxygen, produced from the hydrogen peroxide, reacts with the luminol dianion producing unstable hydroxy hydroperoxide. Latter can decompose with emission of a photon^38^. It is assumed that the gold nanoparticles may interact with the reactants or the intermediates of the luminol-H_2_O_2_ reaction. In particular, it was frequently reported that gold nanoparticles are capable of generating the oxygen-related radicals such as OH^•^, O2^•−^, and other radical derivatives that can accelerate the CL reaction^39,40^.

Until now, applications of chemiluminescence for nucleic acid detection have routinely used enzymes such as peroxidase to generate or enhance the chemiluminescence of luminol^41,42^. The new method overcomes the need of enzymes while providing a better and faster performance.

Gold nanoparticles were conjugated to single-stranded DNA probes (AuNPs-DNA) capable of specifically hybridizing with the complementary regions of the target sequence localized within the genomic DNA of *C*. *fagacearum*. The molecular recognition of the target based on the sequence-specific DNA hybridization leads to immediate aggregation of the AuNPs. In the presence of luminol and hydrogen peroxide (H_2_O_2_), such hybridization results in a significant and measurable change in chemiluminescence intensity.

## Results and Discussion

To demonstrate the concept of nanoparticle aggregation-enhanced chemiluminescence, the fungus *Ceratocystis fagacearum* was used as a model pathogen. *C*. *fagacearum* is the causal agent of oak wilt, a destructive disease of oak trees that causes significant ecological impact, depletion of natural resources, and a growing economic impact^43^.

Rapid and accurate detection *C*. *fagacearum* is essential for disease survey efforts and for implementation of control actions. Because specific antibodies are not available for this pathogen, detection is currently performed by visual diagnostics, fungal isolation and microscopic identification, or nucleic acid detection. Following artificial inoculation, C. *fagacearum* is moves undetected through the transpiration stream of a red oak tree for 2 to 3 weeks without expression of leaf symptoms. Crown wilt is then exhibited, and an infected tree may die within 4–6 weeks of inoculation. Vascular system colonization in species of the white oak group (e.g., Q. macrocarpa, Q. alba) occurs more slowly and the patterns of crown wilt development are different than those in red oaks and may be confused with other diseases. Oak wilt diagnosis in white oak group species is particularly problematic and generally requires laboratory confirmation. While PCR-based detection can be costly (e.g. $120 per sample for one diagnostic laboratory) and requires use of labor-intensive protocols by laboratory-trained personnel, isolation of the pathogen on agar media is time consuming (7–14 days for of the plate growth) and largely ineffective in the dormant season of the pathogen^44,45^. Hence, there is a significant need for a rapid and more cost-effective technique for early diagnosis of oak wilt.

Incorporation of metal nanoparticles such as Au, Ag, Pt, Zn/Cu into a chemiluminescence reaction has been largely shown to enhance the chemiluminescence signal through a combination of different mechanisms that are largely affected by the nanoparticle size^38,46^. Since heterogeneous catalysis is a surface or interface phenomenon, the nanogold catalytic effect increases with the available active surface area of the nanoparticle, which increases with decreasing nanoparticle size^38^. However, the catalytic effect is also affected by the electron density or plasmonic field around the nanoparticles, which reaches its maximum intensity for nanoparticle’s size around 30–50 nm, and decreases for sizes outside that range^38^. As a result, the profile of the catalytic effect of AuNPs on chemiluminescence seems to match the profile of the localized surface plasmon resonance intensity as a function of the nanoparticle size.

In this work, the AuNPs-probe DNA conjugates were prepared using the nucleotide sequences with size of 4.08 nm each. The probe DNA 1 and probe DNA 2 had the length of 12 bases each and given the dimension of a single nucleotide (3.4 Å), the length of each probe DNA was 40.8 Å or 4.08 nm (without impact of terminal SH-groups as linkers for DNA-AuNPs coupling).

Detection of the target was based on hybridization event between two complementary DNA probes (coupled to AuNPs) with the target DNA strand. Binding of the target to the DNA probes leads to the formation of the nanoparticle dimers, in which the strands of DNA probes get attracted to each for the distance of around 8–10 nm. Such distance causes coupled plasmon resonance of AuNPs (plasmon coupling occurs since AuNPs approach each other to the distance shorter than a diameter of one nanoparticle, i.e. 10 ± 2 nm). As it was shown in our previous work^24^, the intensity of electric fields near the particles’ surface decreases with the increase of the inter-particle distance, with an optimum intensity obtained at distances below 10 nm. Existing theoretical and experimental results^47,48^ have shown that assembling nanoparticles give rise to hot spots in the electromagnetic field distribution.

Therefore, the maximum sensitivity of detection in the AuNPs-based systems can be achieved when the target molecule is placed in the inter-particle gap^24^. This gap is the most sensitive region generated by the plasmon coupling of assembled nanoparticle dimers or agglomerates.

At the same time, we did not use shorter nucleotide strands that would decrease distance between AuNPs. Nucleotides should have the proper lengths to provide the specific detection of the target: hybridization of two DNA probes with target strand should eliminate possible interferences with non-targets.

**Figure 1:**
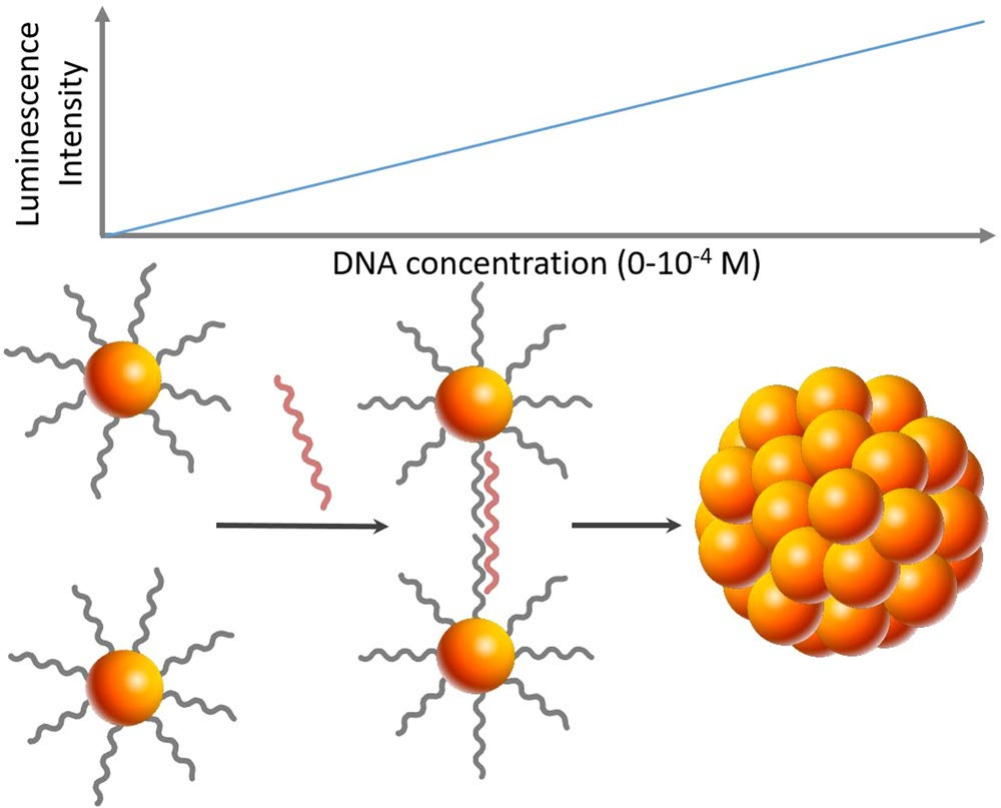
Illustration of the concept of nucleic acid detection using nanoaggregation-enhanced chemiluminescence. The target is added to gold nanoparticles conjugated with two different ssDNA strands complementary to the target sequence: DNA probe 1 and DNA probe 2 (grey). In the presence of the target sequence (red), gold nanoparticles aggregate into nanoballs leading to a significant increase in the chemiluminescence signal of luminol-hydrogen peroxide system (not shown here).

Based on these characteristics, we hypothesized that the assembly or aggregation of gold nanoparticles would also affect the catalytic activity of the plasmonic nanoparticles and thus would induce a change in chemiluminescence intensity. Consequently, detection methods based on nanoparticle aggregation could be easily monitored using chemiluminescence while providing higher sensitivity then colorimetry or UV-visible absorption spectroscopy. Figure 1 shows an illustration of the concept employed in this work for detection of the target DNA where chemiluminescence enhancement originates from the nanoparticle aggregation.

**Figure 2:**
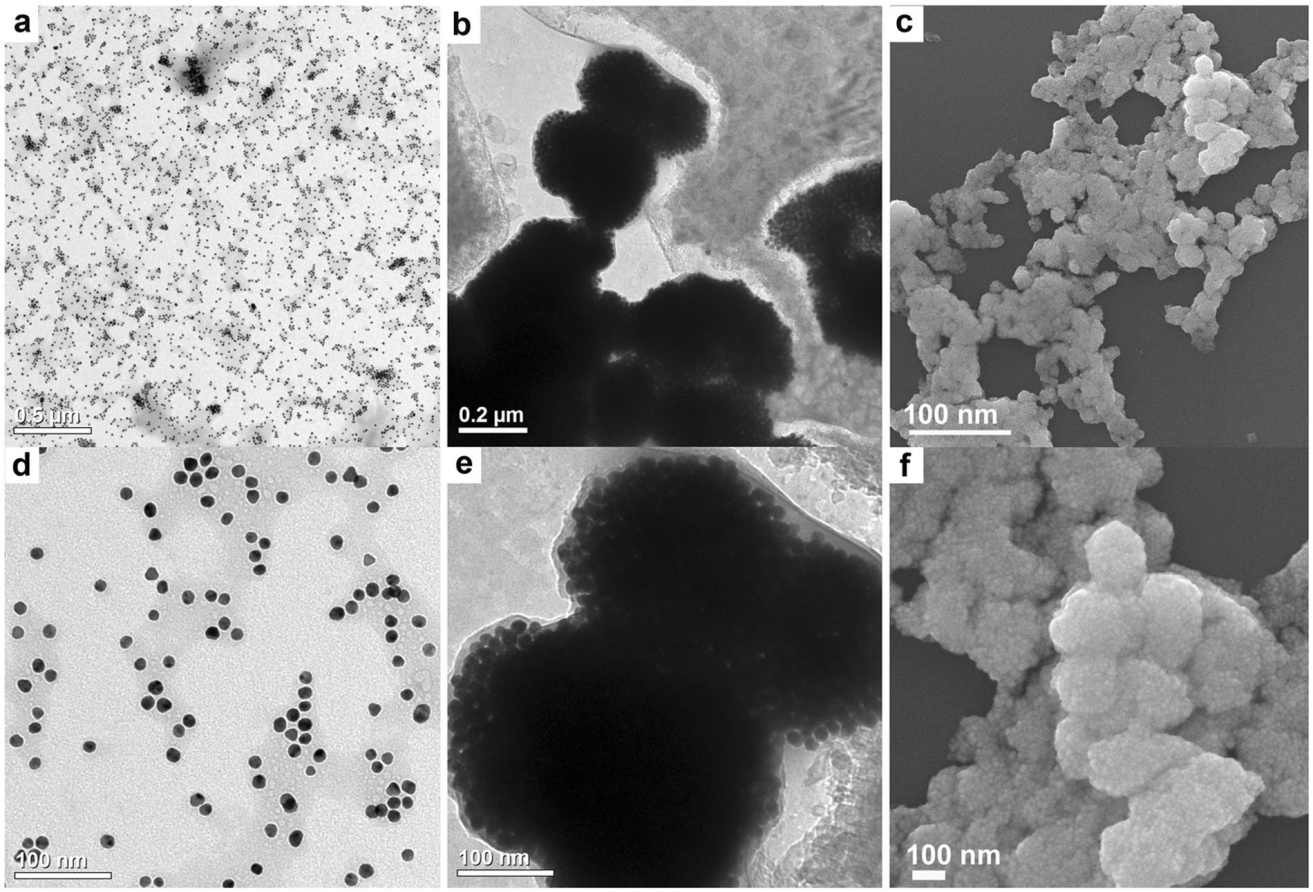
Electron microscopy images of AuNPs conjugated with DNA probes before and after aggregation with target DNA. (**a**) – TEM image of AuNPs-DNA probes (no target DNA added); (**b**) – TEM image of AuNPs-DNA probes in the presence of the target DNA; (**c**) – SEM image of AuNPs-DNA probes in the presence of the target DNA. Images (**d,e and f**) are zoomed views of images (**a, b and c**) respectively.

**Figure 3:**
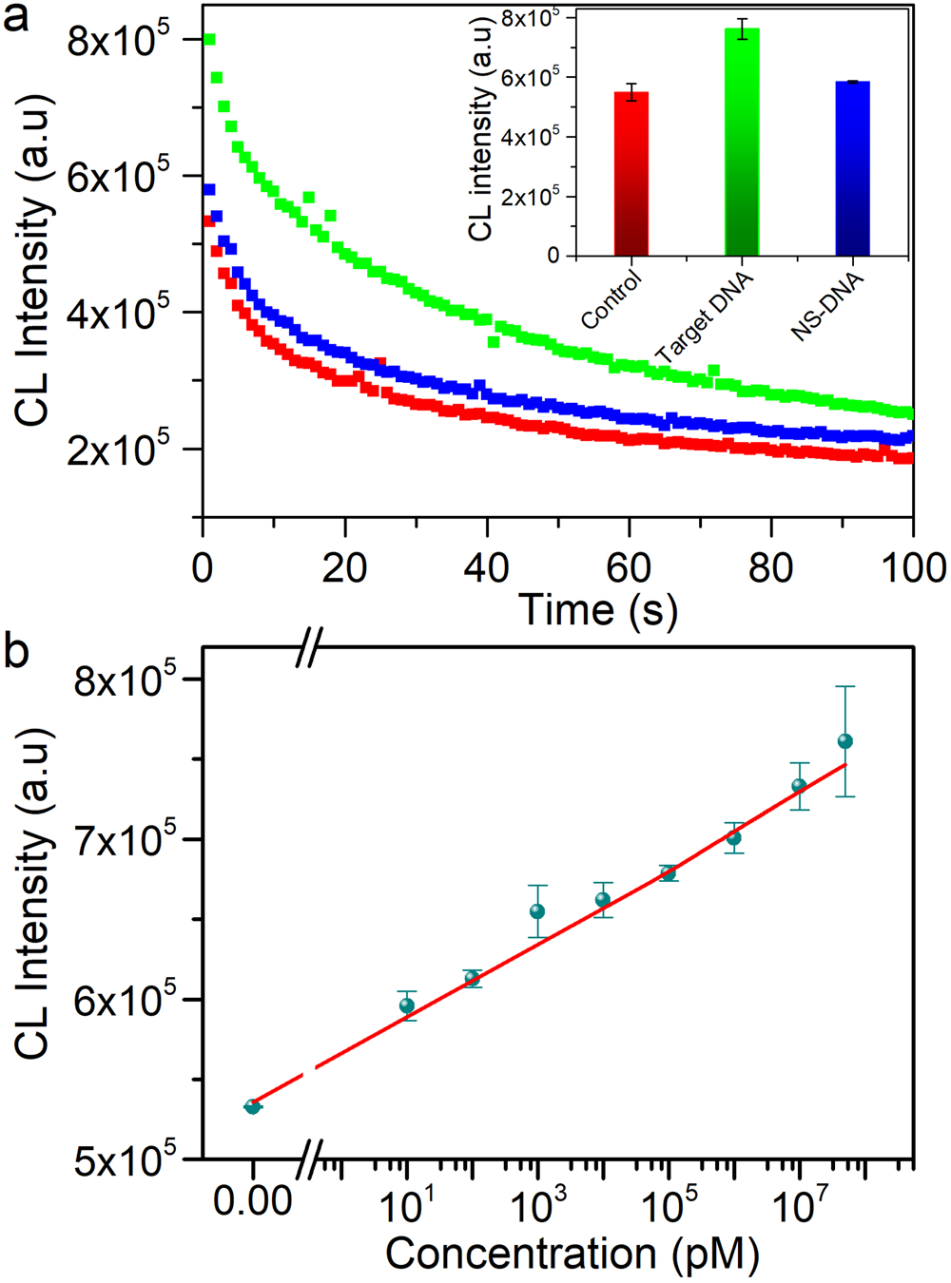
Chemiluminescence detection of *C*. *fagacearum* DNA. (**a**) Variation of chemiluminescence intensity over time for the control sample (red), for the AuNPs-DNA probes after a target DNA (green) and a non-target (NS) DNA (blue) were added. The inset represents the chemiluminescence signal intensity obtained for the above mentioned samples during the first seconds of measurements. (**b**) Linear regression dependence of the chemiluminescence intensity as a function of the target DNA concentration (*y* = 23507.6 *x* + 565141.3, r^2^ = 0.98).

To test our hypothesis, we compared the chemiluminescence of the system luminol-hydrogen peroxide (H_2_O_2_) in the presence of 10 ± 2 nm AuNPs-DNA probes before and after their aggregation caused by DNA hybridization. To prepare the detection probes (AuNPs-DNA probe 1 and AuNPs-DNA probe 2), AuNPs were conjugated with two thiolated ssDNA sequences each of them was complementary to different parts of a target sequence located within the genomic DNA of *C*. *fagacearum*. When the target sequence is added to the reaction medium, the DNA probes recognize and hybridize with the target, leading to the aggregation of the nanoparticles into thin layers or spherical agglomerates depending on the target concentration. The aggregation of AuNPs is confirmed by absorption spectroscopy, transmission electron microscopy (TEM) and scanning electron microscopy (Fig. 2 and Supplementary Figs 1 and 2). The aggregation results in a significant increase in the chemiluminescence signal intensity as depicted in Fig. 3a. The increase in luminescence is proportional to the increase in the target DNA concentration (Fig. 3b), indicating a linear correlation (*y* = 23507.6 *x* + 565141.3, r^2^ = 0.98), with a limit detection down to 260 fM, calculated according to published methods^49^.

In our studies, we explained the correlation between the CL intensity and concentration of the target DNA (Fig. 3b from the manuscript) by relation of DNA concentration in the sample to the degree of nanoparticle aggregation. As described by other authors^50^, assembly of AuNPs has a significant effect on the optical properties of nanoparticles that reflects in excitation of localized surface plasmons of particles. LSPR is, thus, a very suitable and handy tool for real-time monitoring of the assembly process. Plasmon coupling that happen during formation of the nanoparticles agglomerates was also shown by Abbas *et al*.^23^. There are mainly two driven forces for the aggregation of AuNPs to happen: one is the target-guided assembling that brings AuNPs close to each other (cross-linking); and another is salt-induced deterioration of the electrostatic repulsion forces between citrate-stabilized AuNPs (non-cross-linking)^3^. With the use of LSPR spectroscopy, Abbas *et al*.^23^. demonstrated that addition of the linker reagent (p-aminothiophenol or cysteine) to the AuNPs solution makes a second plasmonic band in the UV–visible extinction spectra to appear at higher wavelengths that was due to the plasmonic coupling of assembled nanoparticles. The authors showed that together with shifting over higher wavelengths, the second band also increased in intensity. That spectral change was associated with the progressive aggregation of the nanoparticles into chains and branched network and demonstrated by the authors in SEM and surface enhanced Raman scattering studies. In addition to that, Qi *et al*.^51^. showed that the aggregation of AuNPs was an important effect factor for the catalytic activity of AuNPs on luminol CL system. The catalytic effect of aggregated AuNPs was explained by authors by the possible decrease in their surface negative charge density compared to AuNPs in the dispersed state.

When replacing the target DNA sequence with a random (nonspecific) sequence, no aggregation and no change in luminescence intensity is observed, indicating the specific detection of the DNA sequence from *C*. *fagacearum*. The detection was performed in less than 22 min using a portable luminometer (time not including sample preparation, i.e., DNA extraction from the sample matrix).

To confirm the specificity of the proposed homogenous assay,the C. fagacearum-positive and C. fagacearum-negative samples were analyzed. These include DNA extracted from the pure *C*. *fagacearum* culture (PC), DNA extracted from the wood shavings obtained from the oak trees infected with *C*. *fagacearum* (wood positive; WP), and DNA extracted from the wood shavings obtained from the healthy oak trees (wood negative; WN).

**Figure 4:**
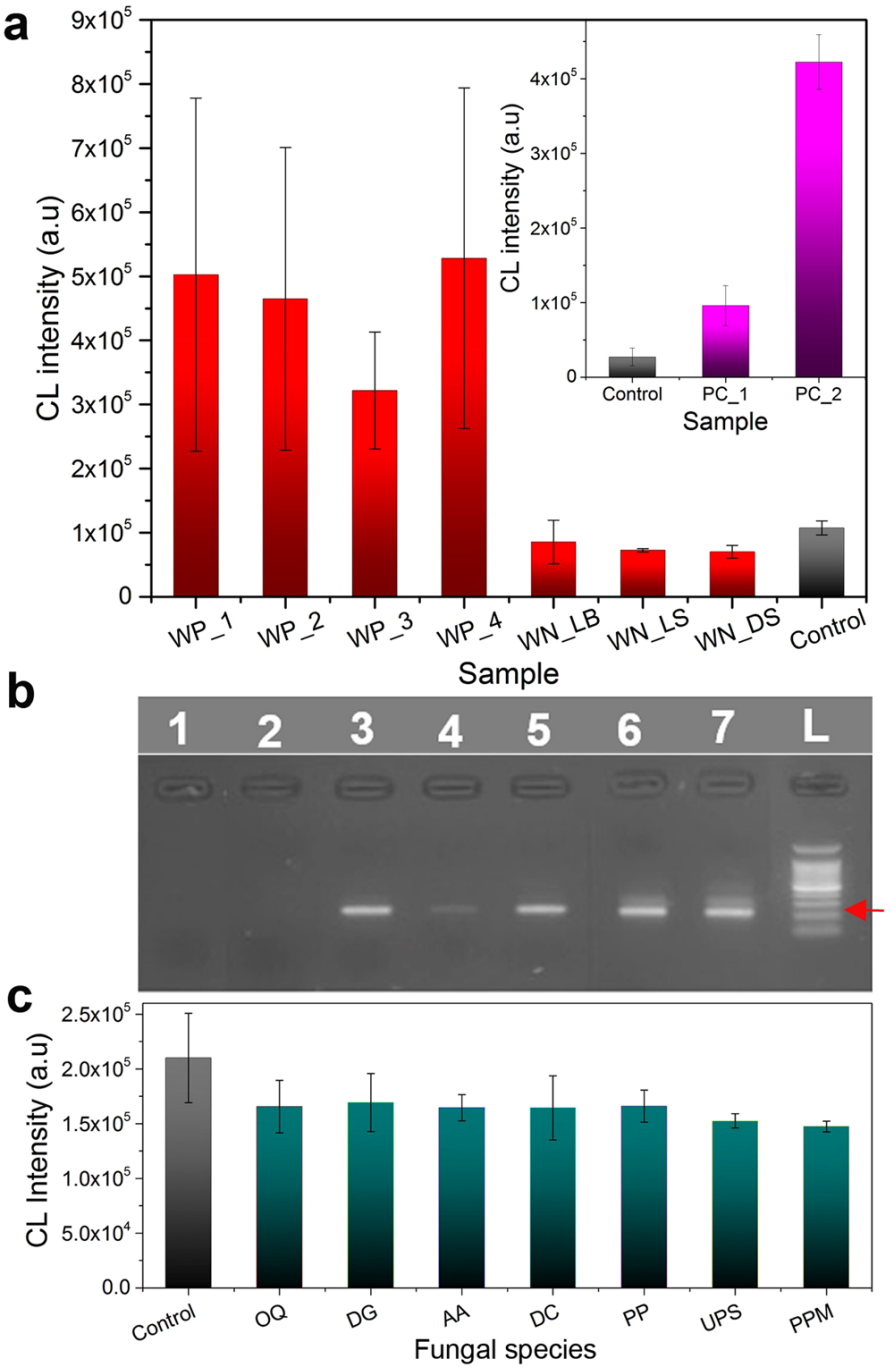
Specificity and selectivity of *C*. *fagacearum* detection with AuNPs-DNA probes. (**a**) A specificity study using wood positive WP (first four columns), and wood negative WN samples (dead stem DS, live branch LB, and live stem LS) and a control (AuNPs-DNA probes with no DNA introduced). Inset shows the CL signals of DNA samples from *C*. *fagacearum* pure culture PC. (**b**) Gel electrophoresis of amplified fungal DNA: 1 – negative control (water), 2 – *C*. *fagacearum* negative wood sample, 3 to 5 – *C*. *fagacearum* positive wood sample, 6 and 7 – pure *C*. *fagacearum* culture), L – DNA ladder. The red arrow indicates the size of a target DNA band. (**c**) Selectivity studies using differentt oak-associated fungi: *Ophiostoma quercus (OQ)*, *Didymella glomerate (DG)*, *Alternaria alternate (AA)*, *Diplodia corticola (DC)*, *Penicillium brevicompactum (PB)*, *Unknown Pleosporales sp (UPS)*, *and Pezicula pseudocinnamomea (PP)*.

First, the DNA sample from pure culture was tested and showed a clear enhancement in the luminescence as compared to the control sample (Inset in Fig. 4a). The control sample was represented by AuNPs-DNA probe 1 and AuNPs-DNA 2 in one system in the absence of any detecting compound. Afterwards, the analysis was performed on the DNA extracts from real-world samples. Figure 4a shows that the *C*. *fagacearum-*positive samples showed remarkably high chemiluminescence signal while the *C*. *fagacearum-*negative samples (obtained from the dead stem (DS), live stem (LS), live branch (LB) of oak trees) showed a weak signal, comparable to that of the control sample.

The samples analyzed by chemiluminescence detection were also subjected to the analysis by nested polymerase chain reaction (PCR) technique to confirm the presence or absence of the target DNA associated with *C*. *fagacearum*. As depicted in the electrophoresis gel in Fig. 4b, the control sample (Lane 1) and the negative wood samples (WN, lane 2) showed no band, confirming the absence of *C*. *fagacearum* colonization. Lane 3–5 containing positive samples (WP) reveal a single band of 280 bp, confirming *C*. *fagacearum* infection. Lanes 6 and 7 shows the same band obtained from DNA extracted from pure *C*. *fagacearum* culture (PC). The band in lane 7 is slightly smeared due to the high concentration of DNA extracted from the pure culture. Thus, PCR detection results were consistent with the results obtained with the chemiluminescence assay.

To evaluate the specificity and the absence of cross-reactivity in the newly developed diagnostic method for *C*. *fagacearum*, we have tested 8 other oak-associated fungi, including *Ophiostoma quercus (OQ)*, *Didymella glomerata (DG)*, *Alternaria alternata (AA)*, *Diplodia corticola (DC)*, *Penicillium brevicompactum (PB)*, *unknown Pleosporales sp*. *(UPS)*, *and Pezicula pseudocinnamomea (PP)*. First, these fungi were identified by isolation and purification of cultures, extraction of DNA, PCR using ITS1F/ITS4 primer pairs, and PCR products sequenced. Following identification, additional extracted DNA was used in detection experiments using chemiluminescence. Figure 4c shows that all analyzed samples exhibited a weak signal intensity comparable to the control sample confirming the specificity of the proposed method to *C*. *fagacearum* uniquely and the absence of cross-reactivity with other oak-associated fungi.

## Conclusions

In conclusion, we report a novel DNA detection method using the enhancement effect of plasmonic nanoparticle aggregation on chemiluminescence. Nanoparticle aggregation is caused by the hybridization of the target DNA with DNA probes attached to gold nanoparticles. The method was demonstrated by the specific detection of *C*. *fagacearum*, the causal agent of wilt in oak trees, and confirmed by isolation on agar and by PCR. The developed method enabled DNA detection at an extremely low level (260 fM). Beside the low cost of the reagents required and the simplicity of the detection procedure, this new method eliminates the need for enzymes and extensive conjugation methods. Furthermore, upon extraction of DNA from the sample matrix, the detection of DNA can be determined within 22 min using a portable luminometer. Once further simplification of DNA extraction steps from real-world samples is achieved, this technique would easily be translated into a rapid, in-field test kit with a hand-held reader.

## Experimental Section

### Reagents

Gold(III) chloride trihydrate, trisodium citrate dehydrate, sodium phosphate saline buffer (PBS), Tris (2-carboxyethyl) phosphine hydrochloride (TCEP), sodium chloride (NaCl), and luminol were purchased from Sigma-Aldrich (USA). Ethylenediaminetetraacetic acid (EDTA) was procured from Boston Bioproducts, USA. Hydrogen peroxide (H_2_O_2_) was obtained from Fluka Analytical, USA. Nanopure water (resistance of ~18.2 MΩ, filtered through a 0.2 µm filter) from a SpectraPure Lab Grade Type 1 DI system (SpectraPure Inc., USA) was utilized for preparation of the desired aqueous solutions (molecular biology grade). All chemicals from commercial sources were of analytical grade or the highest purity available. All the solutions and glassware were autoclaved prior to being used. *Ceratocystis fagacearum (C*. *fagacearum)* and DNA extracted from other fungal strains were collected from the United States Forest Service Northern Research Station, NRS-16, Saint Paul, USA.

Probe DNA for the specific label-free detection of *C*. *fagacearum* were identified from the internal transcribed spacer (ITS) gene region of *C*. *fagacearum* ^45,52^. All oligonucleotide sequences were procured from Integrated DNA Technologies (IDT), USA. The corresponding sequences used in the studies were as follows:

Probe DNA 1: 5′-ACTCAGCAATGA-thio-3′

Probe DNA 2: 5′-thio-TGGTTAAATGCA-3′

Target DNA sequence: 5′-TCATTGCTGAGTTGCATTTAACCA-3′

Non-target DNA sequence: 5′-AGATTGCGATCTCCTGTCCA-3′.

### Synthesis of Gold Nanoparticles (AuNPs)

All glassware used for AuNPs synthesis was cleaned in Nochromix solution followed by Aqua Regia (3 parts HCl and 1 part HNO_3_) according to a standard laboratory procedure. The synthesis of citrate-stabilized AuNPs was based on a modification of Turkevich’s method^53^. Briefly, a 100 mL solution of 1 mM HAuCl_4_ was boiled under stirring and uniform temperature until the formation of bubbles was observed. The solution was then heated for another 25 min. Then, 10 mL of preheated trisodium citrate (38.8 mM) was quickly added to the boiling HAuCl_4_ solution. During this process, the solution turns colorless for a moment followed by a transition from violet to dark-ruby/red. The solution was heated for another 5 min before cooling down to room temperature. The final reddish solution of AuNPs was stored at room temperature and covered with aluminum foil. The size of AuNPs was characterized to be 10 ± 2 nm in diameter using transmission electron microscopy (TEM, FEI Technai T12).

### Synthesis of AuNPs-probe DNA conjugates

All organosulfur modified probe DNA oligonucleotides were received in a disulfide form. For preparing the AuNPs-probe DNA-1 conjugate with thiol as the anchor, the disulfide modified oligonucleotide with a concentration of 50 μM was treated with 100 μM TCEP for 1 h at room temperature to reduce the disulfide bond. This solution was then added to 5 mL of the AuNPs solution, and the mixture was incubated at room temperature for 24 h on blot mixer. Afterwards, 200 μL of 3 M NaCl were slowly added to this mixture followed by incubation at room temperature for 16 h on blot mixer again. The solution was centrifuged at 13,500 rpm for 30 min to separate the AuNPs from the unreacted reagents. The supernatant was replaced with the equal volume of 1 × PBS-EDTA. The same experimental procedure was followed to synthesize the AuNPs-probe DNA-2 conjugate. The obtained AuNPs-probe DNA-1 and AuNPs-probe DNA-2 conjugates were characterized by UV–visible spectroscopy (Shimadzu 1800 spectrophotometer).

### Preparation of fungal cultures

All of the fungal isolates of *C*. *fagacearum* and the other oak-associated fungi (*Ophiostoma quercus*, *Didymella glomerata*, *Alternaria alternata*, *Diplodia corticola*, *Penicillium brevicompactum*, *Pezicula pseudocinnamomea*, and unknown *Pleosporales* sp.) were grown on potato dextrose agar (PDA) (Difco) in 100 mm dia Petri plates for 7 to 14 days at 24 °C.

### C. fagacearum detection via isolation

Standard phytopathological protocols were used to isolate the pathogen from sapwood of branch and/or stem samples from diseased red oaks^54^. Bark tissue was sprayed with 95% EtOH, flame-sterilized and then carefully peeled to reveal outer sapwood areas with vascular discoloration characteristic of *C*. *fagacearum* colonization. Small pieces of discolored tissue were excised using a sterilized wood gouge and then plated on PDA amended with 20% lactic acid (4 ml / l000 ml media). Plates were incubated on the laboratory bench in clear plastic boxes at ambient temperature (~ 24 °C) and standard room lighting for up to 14 days. Resulting fungal growth was either examined directly when pure cultures were obtained or sub-culturing performed to obtain pure isolates. The fungus was identified on the basis of colony appearance and presence of endoconidia^55^.

### Fungal DNA extraction for use in PCR and chemiluminescence assays

Fungal DNA was extracted from pure isolates of the fungi (*C*. *fagacearum*, as well as the other fungi used for specificity testing) growing on PDA and for *C*. *fagacearum* from drill shavings obtained from exposed, discolored vascular tissue of branch or stem samples taken from diseased red oaks^45^. For fungal colonies, DNA for the internal transcribed spacer (ITS) region was extracted per manufacturer’s instructions for a commercial kit (DNeasy Plant Mini Kit, Qiagen). For drill shavings, DNA for the same region was extracted per manufacturer’s instructions for a different kit (QIAamp DNA Stool Kit, Qiagen). Each extraction yielded 200 µl of extracted DNA for use in nested PCR and chemiluminescence assays for detection of *C*. *fagacearum*.

### *C*. *fagacearum* detection via PCR technique

A recently published nested PCR protocol was used to amplify the pathogen DNA resulting from culture and from wood extractions^45^. The first round of amplification was performed with general ITS primers (ITS1F and ITS4) and the second round with specific primers CF01 and CF02 developed by Wu *et al*.^52^. Amplification protocol was per manufacturer’s instructions (QIAamp Mini Stool Kit, Qiagen) as used by Yang and Juzwik^45^. Lysis buffer without template DNA was used for negative controls while extracted DNA from previously identified fungal cultures of *C*. *fagacearum* were used for positive controls for drill shaving samples. Visualization of the PCR product using 2% agarose gel resulted in an amplicon size of 280 bp when *C*. *fagacerarum* was present. Verification was done by sequencing the PCR products and comparing resulting DNA sequences to those in GenBank.

### *C*. *fagacearum* detection via Chemiluminescence technique

DNA specific to *C*. *fagacearum* was detected by chemiluminescence technique using two AuNPs-probe DNA conjugates. CL reaction was conducted using luminol (0.2 mg mL^−1^) and H_2_O_2_ (10 mM). The CL spectra were recorded with GloMax-Multi Jr Single-Tube Multimode Reader (Promega Biosystems Sunnyvale, Inc., USA). Aggregation of AuNPs was also confirmed by microscopic imaging techniques, i.e. field emission gun–scanning electron microscopy (FEG-SEM-JEOL 6500) and transmission electron microscopy (TEM, FEI Technai T12).

## Electronic supplementary material

Supplementary Information
