# Supplementary material for: Rapid and PCR-free DNA Detection by Nanoaggregation-Enhanced Chemiluminescence
Source: Sci Rep. 2017 Oct 25;7:14011. doi: 10.1038/s41598-017-14580-w (PMC5656605; doi:10.1038/s41598-017-14580-w)
Supplement: Supplementary file 1 — Supplementary Information [file 41598_2017_14580_MOESM1_ESM.pdf]

Supplementary Information

Rapid and PCR-free DNA Detection by Nanoaggregation-Enhanced Chemiluminescence

Renu Singh,[†] *Alexandra Feltmeyer,*[‡] *Olga Saiapina,*[†] *Jennifer Juzwik,*[‡] *Brett Arentz,*[¥] *and Abdennour Abbas*[†]*

[†] Department of Bioproducts and Biosystems Engineering, University of Minnesota Twin Cities, MN, 55108-6005, USA

[‡] USDA Forest Service, Northern Research Station, St. Paul, MN, 55108, USA

[¥] Department of Plant Pathology, University of Minnesota Twin Cities, MN, 55108-6005, USA

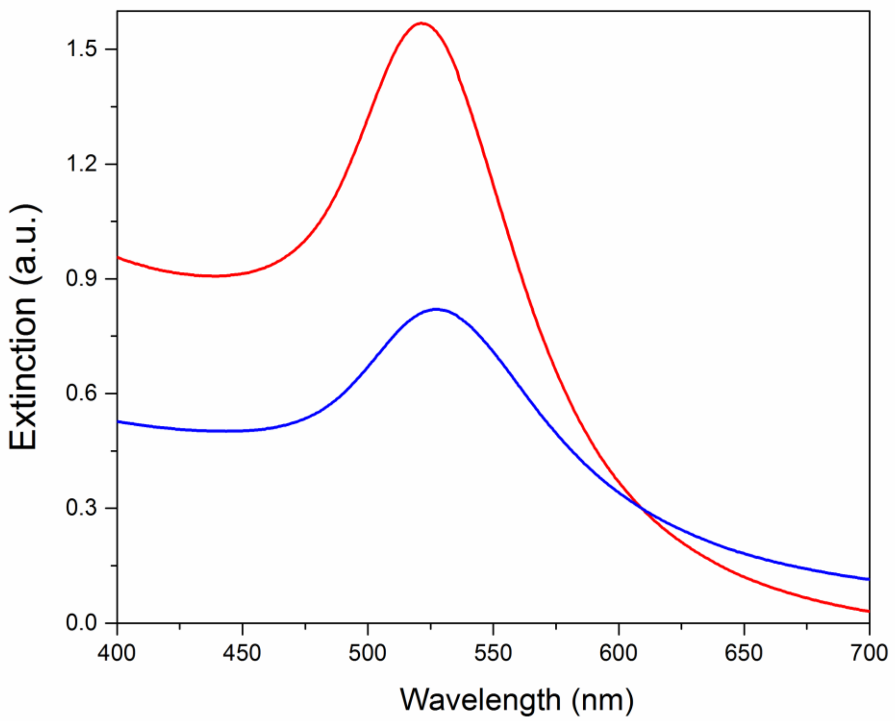

Supplementary Figure 1. Absorption spectra of single AuNPs (red) and AuNPs conjugated with one of the DNA probes (blue). A right-shift in the absorption peak is observed after the conjugation.

TEM analysis

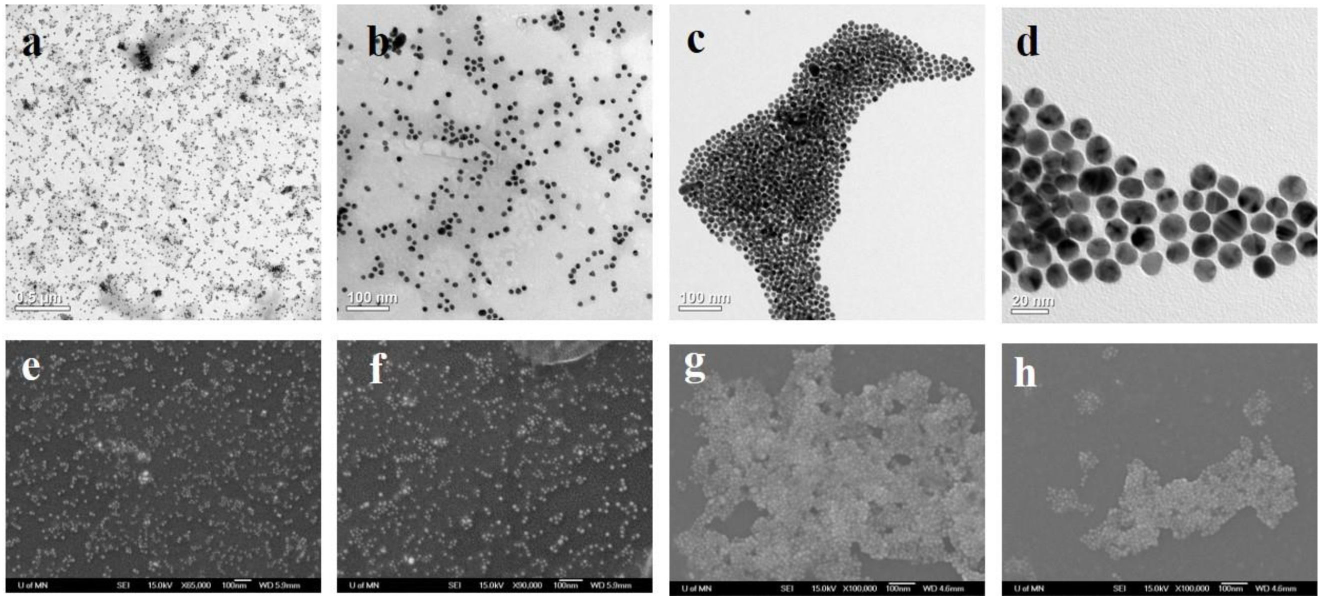

Supplementary Figure 2. Microscopic images of AuNPs-DNA probe conjugates before and after aggregation in the presence of femtomolar concentrations of the target DNA. Top images are from transmission electron microscopy (TEM) showing nanoparticles before (a and b) and after (c and d) addition of the target DNA. Bottom images are from scanning electron microscopy (SEM) showing nanoparticles before (e and f) and after (g and h) addition of the target DNA.
